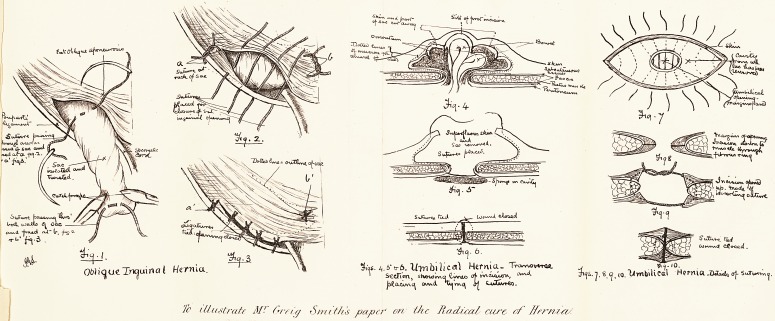# Personal Methods Employed in the Radical Cure of Hernia—Inguinal, Femoral and Umbilical

**Published:** 1890-03

**Authors:** J. Greig Smith


### THE RADICAL CURE OF HERNIA—

### INGUINAL, FEMORAL, AND UMBILICAL.

BY

MR. GREIG SMITH.

In the following paper I desire to record as briefly as possible an account of personal methods employed in the radical cure of hernia. It is no part of my scheme to present well known operations before the reader by way either of comparison or of criticism; my purpose is simply to describe the operations as I have worked them out for myself.

I will say, at the outset, that in my belief the best results in all cases will not be got from a strict and unvarying adherence to any one method. I hold that a skilled and judicious surgeon, familiar with the anatomy of the parts in health and disease, and practically acquainted with the best means of using available tissues for definite purposes, will get the best results. In any given case the details of several methods, selected and combined according as circumstances direct, will often be the best mode of getting a radical cure.

It will be necessary to treat of the leading varieties of hernia apart; but certain details common to all may be generally discussed.

*Prolapse of the Mesentery.*—The elaborate studies of Lockwood (*Hunterian Lectures*, May, 1889) provide us, for the first time, with really trustworthy data for estimating the influence of a prolapsed or elongated mesentery in causing hernia. This influence I regard as less potent even than Mr. Lockwood regards it. Anyone at all familiar with abdominal surgery soon becomes impressed with the fact that, in the living body at least, the mesentery is a very ductile—too ductile—structure, and will permit the intestines to roll out in coils through an opening made in any part of the parietes. We must remember that the mesentery is not mainly or primarily a ligament, but rather a vehicle for blood- and lymph-vessels. The bowels are not all slung up by the mesentery; indeed, a good part of the intestinal canal lies at the level of or above the mesenteric insertion. And, further, we must remember that measurements in the supine posture after death are no criterion of the excursus permitted in the varied postures assumed during life. As a matter of fact, it is when the pubes approaches the sternum and the anterior abdominal wall is shortened, as in lifting weights, that hernia is most frequently produced. In this position no mesentery is short enough to prevent the bowels, which always lie in contact with a possible hernial opening, from finding their way through it. For inguinal and femoral hernia, it is true that the bowel lowest down—that is, generally speaking, the bowel with the longest mesentery—will first find its way through a hernial opening; but it does not follow that a long mesentery of itself, to any practical and estimable extent, predisposes to hernia.

An old intestinal hernia has of necessity a long mesentery; but I believe that to treat it wholly or partially by shortening the mesentery is a surgical mistake. For, in the first place we cannot deny to the mesentery the capacity which every fibrous tissue has of adapting itself in length to the distance it has to traverse; in other words, that if the bowel is kept reduced for a few weeks, it will become adaptively shortened. Here surgical shortening of the mesentery is a superfluity. And if in addition the ductility of the living mesentery be allowed, which can scarcely be doubted, we must admit that to closure of the hernial opening, and to this alone, must we look for an efficient radical cure.

*The treatment of herniated omentum.*—As it is universally agreed that omentum in a hernial sac ought to be removed and not reduced, no arguments in support of the practice need here be adduced. One point, however, demands emphatic attention and that is, the tendency to hæmorrhage which severed and ligated omentum shows on being returned to the abdomen. A surprisingly large number of examples of this accident have been recorded. I have had two in my own practice, fortunately detected and checked in time; and the experience has impressed me so forcibly that, as far as known surgical expedients can provide against the accident, I am determined never to have another.

The explanation of the bleeding after apparently satisfactory ligation is, that the centre of the stump, composed of folds of slippery omentum, retracts, and the compression exerted by the ligature is thus inefficient. Several precautions may be taken. One is, to cut away the omentum beyond the ligature before the ligature is tightened; it is nearly always possible to pull the ligature more tightly after the distal portion is cut away. Another precaution is, to discard catgut and to use only silk. Catgut thick enough to bear much strain does not sink readily into the tissues; if thin enough to sink into the tissues, it will not bear sufficient strain. Besides, it is comparatively elastic. Silk should be used: it ought to be placed as several interlocking ligatures, each ligature enclosing a small piece of omentum; and the ligatures should be tightened after the distal portion has been cut away. Finally, a catch-forceps should be placed on the stump and let slip inside the abdomen with it; and the stump should be re-examined at the last moment, before closing the wound.

Omentum in large irreducible herniæ may, as is well known, go on growing in the sac, and sometimes attains to enormous dimensions. At the neck, however, there is always a sort of pedicle, comparatively free from fat, and here ligation is made. If the fatty tissue is abundant at the site selected for ligature, compression between the blades of large pressure-forceps will make a groove or sulcus, free from fat, in which the ligatures may be placed. It is often possible, even where the omentum seems to be a round cord, to open up folds of thin tissue through which the ligatures may be carried either by a blunt aneurism-needle or a sinus-forceps.

Some surgeons have recommended that the stump be fixed in the hernial opening. This practice I cannot endorse. It prevents perfect closure of the opening, acts as a sort of guide to the weak part of the wall, and provides a band, fixed at both ends, over which intestines may become obstructed. An example of internal obstruction thus produced after herniotomy I have had to treat by abdominal section in the Bristol Infirmary. The ligated stump should always be returned into the abdomen.

**Inguinal Hernia.**—For the radical cure of inguinal ruptures two measures are, I believe, of prime importance:

(1) The utilisation of the sac so as to form a barrier *across* the inner opening.

(2) The closure of the hernial opening, with, where possible, a restoration of the valvular form of the canal.

*Treatment of the Sac.*—To remove the sac is, I believe, to remove a tissue, which may be converted into a most potent factor in the cure; in the case of very large herniæ, perhaps the most potent factor. To divide it at the neck and leave the scrotal portion *in situ*, as in Barker's method, is certainly a saving of labour, but it is a waste of means. In Ball's method of twisting, the loss of the sac is partly made up for by the increase of inflammatory thickening and the tightening-up of loose peritoneum which results from the twisting process. The superiority of Macewen's method to all others hitherto published depends, in my belief, on the fact that he utilises skilfully the sac as a barrier inside the hernial opening. Indeed, I doubt whether some of the subcutaneous methods, such as Wood's or Spanton's, which do not involve removal, would not compete favourably with any of the open methods in which the sac is removed.

Had Macewen's method been brought out before I had tried the method to be described, it is likely that I

should have at once adopted it. Now, seeing no reason for departing from my own method, I might venture on the criticism that the folded sac lying opposite the hernial opening might tend to act as a wedge and force the canal open again. Macewen, indeed, makes this criticism himself on his method. The coherent lump does not seem to be placed at the best mechanical advantage for preventing excursus of the bowels. Re-opening of the canal would seem to me to be best prevented by laying the sac across the opening, its extremities resting on and being fixed to the powerful tissues which lie beyond.

This is the most important feature of the method I advocate: the placing across the ring of the whole sac, or as much of it as may be necessary, and fixing it at the neck and the fundus by sutures passed through the parietes. As a simple and efficient means of bringing the walls of the sac together, and assuring the maximum amount of inflammatory adhesion, I recommend twisting of the sac.* Further, by twisting, it is possible to effect peritoneal closure much higher up than by any other method.

And now to describe an ordinary operation. I may say at once, that if the hernia is of the sort for which I advocate operation, it will not be an easy one. In the case of strangulated hernia, where the proceeding is carried out as a sequence to the main effort of relieving the strangulation, it is usually a very simple proceeding. But in large, or enormous, irreducible herniæ which totally incapacitate for work, or often show signs of

* The claim of priority of method is one which has always very little weight with me. I may say, however, that twisting the sac had been employed by me on a good many occasions before Professor Ball's method was introduced to the notice of the profession.

obstruction—and these are the cases I have chiefly had to deal with—the proceeding is one which may properly be classed among the grand operations of surgery. Here it is not part of my purpose to discuss the selection of cases for operation: I would simply say that I should refuse operation to no case of hernia, however large and however complicated, provided the abdominal cavity is large enough to hold the hernial contents. It is by these bad cases that a method must be tested; almost any method will give a fair per-centage of cures in simple cases.

An incision of suitable length, from two to four inches or more, is made over the neck of the sac, as in ordinary herniotomy. It is, however, carried higher up the abdomen, and at the upper end may often with advantage be curved outwards a little way. The neck of the sac is first isolated; this is done first that the position of the spermatic cord and vessels may be determined, and avoided in all future steps. Where it is possible to do so, the next step should be to hook the finger around the neck of the sac, pull it forwards, and by a little manipulation to turn the scrotum inside out. The whole hernia is now on view, and is carefully surrounded with warm antiseptic sponge-cloths. The sac is now isolated by dissection and tearing from above downwards, the cord being carefully freed and left with a full share of areolar tissue around it. If there is doubt at any point as to the limits of the sac, it should be at once opened, and the finger inside made to serve as a guide. If the hernia is reducible, it is returned when the sac has been isolated; but before twisting the sac an opening should be made at its fundus, through which the finger is inserted to make certain that the hernial contents are completely returned.

If the hernia is irreducible, the sac is laid freely open upwards and downwards by scissors through an opening that has been made at a part where the contents are not adherent. To avoid points of adhesion to underlying parts, the incision may have to be guided in lines that are not straight. To give additional room, and to minimise the bleeding from adhesions, it is usually best at once to ligature omentum at the neck where it is free from adhesions, divide it and at once return the stump with a long catch-forceps temporarily attached to it; on the distal side a forceps also is placed.

Then the subsequent steps of liberating adherent bowel and returning it, or of separating omentum from sac or bowel, or both, are proceeded with as circumstances direct. Some adhesions may be sponged apart; others require tearing and forci-pressure; while others demand ligation. The field of operation should be kept warm and isolated and clean by frequently changed sponge-cloths or large flat sponges.

All bowel being returned, and all omentum having been removed from the interior of the sac and all bleeding having been checked, the fundus of the sac is grasped by two pairs of forceps, which are handed over to an assistant, who holds them well to the inside or the outside as the case may be, and rotates them as he is directed, thus twisting the sac. By turning the sac to one side or the other, and pulling more on one forceps than another, the peritoneal opening, may be closed at one or other side of the hernial opening as we may desire, and not opposite to the opening. The assistant having given two or three turns to the sac, the forefingers, behind (feeling the cord) and in front, tease open the areolar tissue separating the neck of the sac from the inner parietes as high up as may seem desirable. Twisting, by gathering together the walls of the sac, very materially aids this process of separation. If the sac is exceptionally long, a piece of its fundus may be cut away. If it is very thin, it should not be twisted very tightly, in case vascular stasis and necrosis result.

The twisted and fully isolated sac now lies in the ring, with forceps attached to its fundus. (Fig. 1.) Silk ligatures, with needles at both ends, are now placed, one ligature at the fundus, another at or above the neck of the sac. The ligature at the fundus is made to pass several times through both walls of the sac. The ligature, or rather suture, at the neck is made to gather together a sufficiency of areolar tissue on the sac. There is no strong objection to its perforating the sac; but it should not be made to surround it, on account of the risk of sloughing.

The sac is now laid across the opening, as nearly transversely as possible, and fixed by the sutures at its extremities to the firm parietes beyond. In the drawing (Fig. 2) the fundus of the sac is represented as being fixed on the inside; but as often as not the reverse position is advisable. The aim is, to get the internal orifice of the twisted sac opposite the strongest part of the parietes, and away from the internal opening. As a general rule, it is best to twist in a line opposite to that of the axis of the canal. But no absolute rule is possible; each case must be dealt with according to its peculiarities. By means of the curved needles, held in a needle-holder, or by a handled needle like that of Macewen, the sutures are passed through the parietes under the retracted skin. The position of the deep epigastric is first ascertained and avoided. It is usually best to fix the fundus first. Supposing, as in the figure, the fundus is paced to the inside, the forefinger of the left hand is carried upwards under the conjoined tendon, carefully separating the peritoneum. The finger acting as a guide, the needles are made to pass through the conjoined tendon and aponeurosis of the external oblique; or, if the hernial opening is large and the sac not small, through the rectus. They are made to take a good hold of the tissues, the sutures being at least half an inch apart. During this manœuvre the skin and superficial fasciæ are drawn inwards. The forceps are removed and the sutures pulled tight, while the attached fundus is guided into position. The sutures are now tied.

The forefinger of the right hand is now carried under the peritoneum on the outer and lower side of the opening, separating the peritoneum in the same way, and the sutures at the neck are introduced as far outwards and downwards as possible. If possible, they should be made to grasp Poupart's ligament and such fibres of the internal oblique and transversalis as may be within reach. When these sutures are tied, the sac is drawn completely inside the canal and lies obliquely across it. It fortunately happens that a large hernial opening is usually associated with a large sac, so that the available tissues are provided in necessary quantity. The dimensions of the sac must, however, be taken into account in fixing on the points for placing the sutures.

In small or even in moderately sized herniæ it is not necessary to place a fixation-thread at the neck if the twisting is made to begin well to the inner side of the internal opening. This thread may be omitted also if the sac is very thick and the areolar tissue dense.

The inguinal canal is now closed. (Figs. 2 and 3.) Macewen's method of closing the internal ring is recommended. But in very large herniæ, in which the edges of the opening are surrounded by thick areolar—almost cicatricial—tissue, it may be impossible without dissection to isolate the conjoined tendon or the fibres of the internal oblique and transversalis. This dissection may be done and the layers caught; but I believe it will be found sufficient to push the points of the needles obliquely upwards through the internal pillar, taking in as much of the deep muscular layers as possible. On the under aspect of the opening the needles are passed, where possible, under Poupart's ligament, which is raised forwards out of danger by the forefinger. The cord meanwhile will have been observed to be out of danger. Three, four, or five sutures, according to the size of the opening, will be sufficient to close it. The fibres of the external aponeurosis will be crowded together (as in the diagrams, Figs. 1 and 2) and adherent. If it is difficult to close the opening, a few incisions made along the fibres will free them; and when the sutures are pulled tight, the opening is easily closed and their parallelism restored. (Fig. 3.)

In congenital hernia certain variations in procedure are essential. The sac is completely divided just above the testicle, leaving enough tissue to form a tunic proper to the gland. A few sutures may serve to fix this tunic in proper position. If, as is not uncommon in these cases, the cord is firmly adherent to the sac, or lies in a special sulcus, then it is best to cut the sac completely away from the cord, leaving a long strip of sac attached to it. Division is carried well inside, and the sac may be twisted and otherwise dealt with as if it were entire. In some congenital herniæ there is a small neck, and perhaps a large mass of omentum in the sac. In three of these I have cut the sac into two long flaps or aprons, and fixed the extremity of each flap on opposite sides of the hernial opening withont twisting. The result has been equally good.

The cutaneous wound is closed over a drainage-tube carried to the bottom of the wound on the abdominal aspect. Pressure over the ring induces œdema of the scrotum; a simple absorbent dressing, fixed with strapping, is sufficient. The scrotum rests elevated on a pillow of wool laid over a broad piece of strapping fixed to the top of the thighs.

At the end of three weeks or thereabouts the patient is permitted to get up and walk about. The wearing of a truss I believe to be detrimental. A truss undoubtedly causes atrophy of the tissues it presses upon. In the face of all surgical experience that elastic pressure is one of our most potent means of causing absorption of inflammatory thickenings, it is idle to argue that a truss will not do so here. Nothing causes a chronic indurated bubo in the groin to melt away so quickly as the wearing of a truss over it; and if we want our traumatic inflammatory thickening to remain strong and dense as long as possible, the wearing of a truss is the last measure we should adopt. To have to make the patient wear a truss at any time after operation is tantamount to a confession of failure in the operation—partial, and perhaps complete.

**Femoral Hernia.**—In this variety of hernia I use the same method of placing the twisted sac across the opening—only that no attempt is made to close the opening, and the neck is not fixed. One side of the sac is pulled well down, and torsion made in the opposite direction. The fundus of the sac is attached either on the outside above Poupart's ligament, or directly above the ring, or to the inside. I have never had to perform the radical cure on a femoral hernia that was not strangulated; and I have seen only one such operation, by one of my colleagues. The proceeding is extremely simple, there being no cord to consider, the opening always being small (except in some cases that have been operated upon for strangulation), and the sac in the majority of cases easily isolated.

**Umbilical Hernia.**—The operation which I recommend for umbilical hernia is, to remove the whole sac, with all superfluous cutaneous and fatty tissues, and to bring the wound together exactly as in an ordinary abdominal section.

A large umbilical hernia in a stout woman (which is the hernia we are usually called upon to treat) is always a complicated affair. It will contain bowel and omentum, not in one simple sac but in many recesses, partially separated by thick septa, and the contents, septa and sac, matted together by old and dense adhesions. These recesses burrow under the skin in the abundant fatty and areolar tissue in all directions. Irreducible omentum may attain to enormous dimensions in such a sac, while its pedicle may remain small: it derives nourishment from the numerous adhesions which it forms to the sac as well as from its pedicle. The pedicle of the omentum is naturally, in most cases, on the upper aspect of the umbilical opening and the bowel below it. Bowel may be found coiled up in recesses at any part of the sac, adherent or non-adherent.

Usually at the most prominent part of the tumour there is a piece of very thin skin, freely movable over the hernial contents; the first incision is made vertically through this. (Fig. 4.) The bowel or omentum being exposed, the incision is prolonged upwards and downwards to the extreme limits of the sac by scissors guided by the forefinger.

Through the hernial contents we now seek for the hernial opening. Any reducible bowel is at once returned; and if it seems easy to liberate the whole bowel by separating a few adhesions, this may at once be done and the whole pushed through the opening. If the intestinal adhesions are dense and abundant, it is best to proceed at once to the next step; namely, ligature and division of the pedicle of the omentum. The pedicle is carefully unravelled, to make certain that no bowel is embedded in it, and the ligatures applied in the manner already described. A catch-forceps prevents bleeding from the distal half. It is unnecessary to separate omentum from sac, as the whole is to be removed when the bowel is completely returned. A flat sponge placed inside the umbilical opening prevents protrusion of bowel and collects any extravasated blood.

All the thin superfluous skin is now removed, along with the adherent underlying sac; and this may be done freely (see Figs. 4, 5, 6, 7), for the surrounding skin is usually very elastic and easily stretched. The rest of the sac, with its contained and adherent omentum, is removed (Fig. 4) by free incisions, rather than an elaborate dissection, down to the umbilical opening. If the umbilical opening is not large and its surrounding fibrous tissue not very dense, it may at once, after its edges have been pared, be closed by single sutures carried through the skin under the areolar tissue, which marked the limits of the sac, and through the edges of the ring in the manner shown in Figs. 5 and 7. When the sutures are tied, the wound in section then appears like one closed after an ordinary median abdominal section. (Fig. 6.)

But if the opening is large, or its margins dense and cicatricial, then the use of a large flange-stitch (Figs. 8, 9, 10), either separately or in conjunction with the general suture, is advisable. The cicatricial tissue is divided all round the opening at its free margin down to the rectus muscle, whose margin may bulge well into the wound. The suture is made to pass, first through the upper fibrous edge, then through the rectus, then through the lower fibrous edge of one side; and in reverse order through the tissues on the other side. (Fig. 9.) When closed, the wound should present the appearance shown in Fig. 10. In order to strengthen the umbilical opening as much as possible, and also to exert vertical traction on it, the parietal sutures are placed as shown in Fig. 7. Even if the umbilical opening is separately sutured, it is better to carry the cutaneous sutures at least a little way into the muscle.

The sutures are all placed in position before being tied, and the sponge is removed before tying them. I would suggest as a final proceeding that the finger be passed inside and made to examine the parietes all round the opening, and especially directly above and below it. This is to make certain that there are no small herniæ through the linea alba. By doing this in one case, I avoided what might have been a catastrophe. The patient, under the care of Mr. Kinneir of Malmesbury, had several times had symptoms of obstruction, almost of strangulation, in the sac. I operated in one of these attacks. To our surprise, no evidence of strangulation was found in the abundant bowel which protruded, and the operation as above described was carried out; the sutures were inserted, and the sponge removed. Omentum, sac, and fat removed (the lady was very stout) nearly filled a small washing-basin. Still feeling doubtful as to whether the strangulation had been removed, I inserted my fingers into the cavity, and found a small knuckle of bowel passing through and firmly gripped in a hernial opening about two inches above the umbilicus. The incision in the skin was prolonged upwards till it was exposed, the sac was opened, and the bowel was found distinctly strangulated. The opening was nicked at the upper margin of the opening, and the bowel fell back. A couple of stitches closed it. Another case, under the care of Dr. Davis, of Clevedon, had several—at least two—openings besides the umbilical one. This one was complicated by the presence of a very large—partly calcified, partly cystic—fibroid tumour of the uterus, which hung down over the thighs, and had to be removed. The hernia, it need scarcely be added, was not a simple one to deal with.

In the case of umbilical hernia I do not advise the utilisation of the sac, for two reasons: firstly, because it is not wanted, as there is abundance of thick, strong tissues without it; and secondly, because I should dread its sloughing, as it must be separated from its chief source of vascular supply.

Certain details in each case may properly be varied. Thus, in a very stout woman, I have removed a wedge of fat along each side of the opening, and brought the parts together without attempting to carry the suture to the extreme depths of the wound. In a circular opening, with dense, almost cartilaginous, margins, an incision may be made into the ring all round, so as completely to liberate the recti and render it possible for their margins

Fig. 1.

J.G.S.

Fig. 2.

Fig. 3

Oblique Inguinal Hernia.

Fig. 4

Fig. 5

Fig. 6.

Figs. 4. 5 & 6. Umbilical Hernia – Transverse section, showing lines of incision, and placing and tying of sutures.

Fig. 7

Fig 8

Fig. 9

Fig. 10.

Figs. 7, 8, 9, 10. Umbilical Hernia. Details of suturing.

*To illustrate Mr. Greig Smith's paper on the Radical cure of Hernia.*

almost to fall together. The fibrous edges of the ring may then be ignored. After all, there is no barrier against hernia so efficient as muscle.

The cases I hope to relate in a future paper. Suffice it here to say that the results thus far, extending over a period of six years and embracing about thirty cases, in the hands of my colleagues and myself, have been such as fully to warrant the operations being recommended to others.

## Figures and Tables

**Fig. 1. Fig. 2. Fig. 3 Fig. 4 Fig. 5 Fig. 6. Fig. 7 Fig 8 Fig. 9 Fig. 10. f1:**